# Genomic leftovers: identifying novel microsatellites, over-represented motifs and functional elements in the human genome

**DOI:** 10.1038/srep27722

**Published:** 2016-06-09

**Authors:** Natalie C. Fonville, Karthik Raja Velmurugan, Hongseok Tae, Zalman Vaksman, Lauren J. McIver, Harold R. Garner

**Affiliations:** 1 Via Bioinformatics and Clinical Genetics Network, Edward Via College of Osteopathic Medicine, 2265 Kraft Dr, Blacksburg, VA 24060, USA.

## Abstract

The human genome is 99% complete. This study contributes to filling the 1% gap by enriching previously unknown repeat regions called microsatellites (MST). We devised a Global MST Enrichment (GME) kit to enrich and nextgen sequence 2 colorectal cell lines and 16 normal human samples to illustrate its utility in identifying contigs from reads that do not map to the genome reference. The analysis of these samples yielded 790 novel extra-referential concordant contigs that are observed in more than one sample. We searched for evidence of functional elements in the concordant contigs in two ways: (1) BLAST-ing each contig against normal RNA-Seq samples, (2) Checking for predicted functional elements using GlimmerHMM. Of the 790 concordant contigs, 37 had an exact match to at least one RNA-Seq read; 15 aligned to more than 100 RNA-Seq reads. Of the 249 concordant contigs predicted by GlimmerHMM to have functional elements, 6 had at least one exact RNA-Seq match. BLAST-ing these novel contigs against all publically available sequences confirmed that they were found in human and chimpanzee BAC and FOSMID clones sequenced as part of the original human genome project. These extra-referential contigs predominantly contained pentameric repeats, especially two motifs: AATGG and GTGGA.

In April of 2003 the Human Genome Project was declared complete, and from it we gained a framework to build the reference genome upon which the majority of analyses are anchored. The scope of the Human Genome Project was focused on the 94% of the genome that is euchromatin^1^, now sequenced to 99% completion^2^. Attempts are being made to complete the 1% of the incomplete “complete” human reference^3^, however we and others have hypothesized that some genomic sequence regions (which may contain functional elements, genes) may be missing from the human reference because they are embedded in refractory repetitive DNA sequence, e.g. microsatellites (MSTs)^4^. MST sequences, regions of repeated 1- to 6-mer DNA motifs, are abundant throughout the genome and are a source of significant genomic variation^5^. However, to date, analysis of microsatellite-containing loci has been limited because standard exome enrichment and whole genome sequencing uses software to mask out repeats^6^, focuses on capturing non-repetitive DNA, or is designed to capture only a small subset of the known MST loci^7^. In this paper we present a novel target enrichment strategy specifically designed to enrich for all microsatellite loci based on the repeat motif, rather than the flanking sequence, as baits, and have paired this technique with our recently developed method for analysis of unmapped reads^8^. Our analysis has revealed: 1) assembly of contigs from unmapped genome sequences and high-depth sequences from this novel target enrichment system that specifically selects for repetitive elements enables the quantification and characterization of these regions; 2) concordant contigs, those that appear in multiple samples, contain new structural elements (potential genes/pseudogenes, etc.), a subset of which have high similarity to expressed mRNAs; 3) these extra-referential genome regions are dominated by 5-mer repeats, in particular, an AATGG and a GTGGA centromeric repeat. This platform technology has the potential to extend “reference genomes” and identify new functional elements.

## Methods

Standard exome enrichment sequencing is designed using a bait set that contains the sequence of the known high complexity exomic regions. However, portions of the human genome remain unknown and as such are not captured and evaluated by current enrichment technologies. In addition, whole genome sequencing, which can be used to sequence these additional unknown regions is limited in its ability to evaluate these regions because sequencing reads are aligned to the known reference genome, and they lack sufficient sequencing depth for reliable assembly. Although these methods (WGS and exome enrichment) are excellent for evaluating a large portion of the genome, they are not optimal for identifying and aligning novel genomic sequence (i.e. gap filling, finishing genomes containing highly repetitive regions). Similarly, only reads in RNA-Seq data that are aligned to known reference genes are quantified, thus, an incomplete reference genome also impacts expression studies. One potential reason that sections of the human genome remain unknown, or are not included in the reference, is that they contain highly repetitive DNA that makes it difficult to sequence and align properly. We have created a reference-independent enrichment method that is designed to specifically enrich for repetitive DNA. This global microsatellite enrichment (GME) assay uses a bait design in which each 120 nt bait is composed of 4 × 30 nt segments, selected to minimize the potential for intra-bait hairpin formation. Every possible 1–6 nt repetitive motif is represented within the bait set.

Design of global microsatellite enrichment (GME) bait set: We designed a custom bait set that target all 1–6-mer microsatellite motifs. Each 120 nt bait is broken into four 30 nt regions, each of which targets a different motif sequence. We programmed and ran a custom PERL script to design the baits to maintain approximately a 40% G/C content along the full length of the bait (across all four motifs on each bait). The custom script also evaluated the potential for hairpin formation of the baits and selected motifs for each bait to have a lower probability of internal hairpin formation. The baits were uploaded into Agilent’s eArray.

Enrichment and Sequencing: Agilent exome enrichment, and our custom enrichment were performed according to the manufacturer’s directions. For the combined GME + Exome enrichment, the bait sets were combined in house. All enrichments were sequenced using the 150 bp Illumina HiSeq Rapid-Run.

Cell culture: DLD1 (ATCC^®^ Number: CCL-221™) and SW403 (ATCC^®^ Number: CCL-230™) human cell lines were purchased from ATCC ( http://www.atcc.org Date of access: 01/06/2014). Cells were grown to confluence in DMEM + 10% FBS at 37 C with 5% CO_2_ (DLD1) or Lebovitz media at 37 C with no CO_2_ (SW403). Genomic DNA was isolated using Qiagen DNA Blood and Tissue kit according to the manufacturer’s protocol. DNA for the 16 normal samples was purchased from Coriell.

Novel microsatellites prediction from unmapped reads: Sequenced reads of all samples were obtained in the form of fastq files. The sequenced reads were paired-end with a sequence length of 150 bases. The reads were quality checked and trimmed using Trimmomatic. The program uses sliding windows to check for the quality and a window of 10 bases was used. A quality score threshold of 20 was used; a 20 quality score means an error probability of 1 in 100 bases. The length threshold was set to 70 bases so any sequence read that is shorter than 70 bases after the quality trimming will be filtered out.

The BWA aligner was used to align the paired-end reads to the human reference genome, Hg19. A custom written python program was used to extract the unmapped reads from the sam files and output them in fasta format. The SAMTOOLS view and index programs were used to sort the sam files and convert sam file to bam format. The ‘add-read-groups’ program in the Picard software suite was used to add read groups to the bam files. The GATK program was used for indel realignment. The bam file that was indel realigned was then used to collect the unmapped reads in fasta format using a custom written PERL script for further processing.

The fasta file with unmapped reads was passed on to a shell program to check and remove recurring “N”s in the unmapped reads. Any read shorter than 50 bases after the removal of “N”s was discarded. The “N” filtered unmapped reads were used to form contigs using the Velvet program. The kmer length (hash length) used for the velveth program was 71. The choice of an odd number for hash length is a requirement of the program so as to avoid palindromes. The resultant contig file is then passed on to the Tandem Repeat Finder (TRF) program for microsatellites identification. Note that there are a total of 1811360 known microsatellite loci in the Hg19 reference genome as identified by TRF. The match weight, mismatch penalty, indel penalty, match probability, indel probability, minimum alignment score and period size used are 2, 7, 5, 80, 10, 14 and 6, respectively. The ‘-h’ parameter was used to suppress HTML output. A custom written PERL program was used to extract the predicted microsatellite list from binary output provided by TRF. In order to check if there are known microsatellites in the list generated by TRF, the TRF identified microsatellites are BLASTed against known genomes. For this purpose, a PERL program was written to add flanking regions (30 bases on each side) to the microsatellite list by referring back to the contigs file generated by Velvet. The microsatellites with flanking sequences are then BLASTed using the blastn program against the blast formatted nucleotide sequence database and the human genome database downloaded from https://ncisf.org/software-databases/blast-databases (Date of access: 08/09/2014). An e-value of 0.001 was used for the blastn program. A PERL program was written to separate the microsatellites that were either found in the human or the nucleotide sequence database hence leaving only novel microsatellites.

Read number calculation: The read depth is calculated from the contig depth using the formula provided by Velvet. For a given contig the information about the contig is provided in the contig ID by the Velvet program (e.g. NODE_35_length_226_cov_17.079645). Using this information the contig coverage can be converted into read depth: C = Ck ∗ L/(L − k + 1). C is the read depth, Ck is the contig coverage, L is the average read length, and K is the read kmer length used.

Identifying novel contigs/MSTs: Any assembled contig that has 100% identity match with a human or a NT database sequence for more than half the length of the query sequence, then the contig is considered known. The remaining contigs are considered novel. All TRF predicted MSTs in the novel contigs are considered to be novel MSTs.

Kmer calculation: The kmers are divided into six categories: 1-mer, 2-mer, 3-mer, 4-mer, 5-mer and 6-mer. The main input file to calculate kmer frequency information is the final output from the previous section that contains a predicted novel microsatellite list. The kmers were grouped into families. A kmer is added to a family if a) they are cyclically same or b) they are cyclical reverse complements. For example, if AACT is a family name, then TAAC, CTAA and ACTA belong to the same family. According to reverse complementarity, AGTT, TAGT, TTAG and GTTA also belong to the AACT family. So an N-mer family has N*2 possible family members. All the read depths in a family are summed and hence each family has one kmer read depth value. To aid in the comparison of kmer families across samples, the name of the kmer families are kept similar. After grouping the cyclical kmers and cyclical reverse complements kmers, measures were taken to unify the family names across samples. The entire kmer calculation procedure was done using a series of custom python scripts.

Concordant contig calculation: The novel contigs from all the 16 + 6 samples were pooled together to find contigs that are observed in two or more samples, i.e. concordant. The pooled contigs were converted into a fasta file and was formatted into a BLAST database. All the individual contigs were then BLASTed against the database. Alignments that are more than 70% of the query length and aligned with 0 or 1 mismatch (to allow for potential individual variation) were considered for further analysis. A python script was written to generate two lists (with no mismatch and 1 mismatch) of concordant contigs that were found in more than one sample. The contigs in each concordant group were assembled into one contig sequence using the CAP3 program.

Evaluation of contigs for gene-like structure: All concordant contigs were fed into the GlimmerHMM program for Gene-Like Structure (GLS) prediction. The program was trained using the human training data provided with the GlimmerHMM package. The program can predict one or more exons in a given contig. The predicted exons in a GLS can be of three types; initial, internal and final. All the GLS with more than one exon were considered for further processing.

Comparison of putative cDNA to RNA-seq data: The start and end positions of the exons were obtained from the GlimmerHMM output to extract putative cDNA sequences. The cDNA sequences of all concordant contigs from all the samples were combined to make a single cDNA database. Ten lymphoblastoid RNA-Seq samples were obtained in the form of FASTQ files ( http://www.ebi.ac.uk/arrayexpress/experiments/E-GEUV-1/samples/ Date of access: 15/01/2015) and were BLAST searched against the cDNA database. A cDNA query sequence that aligned to an RNA-Seq read with an identity of 100% and with an alignment length longer than 70% of the query length was considered to be a highly similar match. The RNA-Seq reads evaluated in this study were on average 75 bases long. The results of the both the BLAST searches were combined and are provided in Extended Data Table 7. Analysis of the BLAST search output was done using custom written python scripts.

Similar to the paragraph above, the concordant contigs were searched against RNA-Seq sample reads. The results of this BLAST search are provided in Extended Data Table 5.

**Figure 1:**
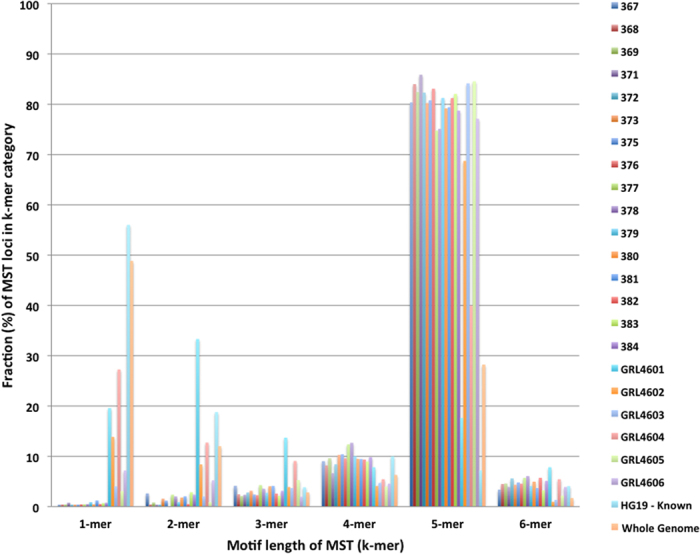
Frequency of MSTs motif classes in unmapped reads relative to those found in the reference genome (HG19) and the whole genome. The majority of novel microsatellite loci captured using our method contained pentameric repeats. For comparison, most loci found in the reference genome are not pentameric; and analysis of whole genome data confirms that much of the missing genome is associated with pentamer repeat regions.

Whole genome analysis: The k-mer analysis graph presented in Fig. 1 contains the k-mer distribution of all the 22 samples along with the human reference genome and a whole genome sample. The whole genome sample was added to compare the k-mer levels of the six samples with a sample where the entire genome is sequenced without enrichment bias and not just the exome.

A B-Lymphocyte whole genome DNA sequencing sample (HG00106) was downloaded from the 1000 genomes project for this purpose. The paired-end sequencing reads were downloaded in the form of a FASTQ file. The reads were N filtered and mapped to the human reference genome (Hg19) using BWA. The unmapped reads were separated from the SAM file. The unmapped reads, in the form of a FASTA file, was used as the input for the TRF program to predict MST loci. The same parameters used for the other samples for TRF was used here too. A custom written PERL program was used to analyze a list of known MSTs and the TRF output data file to generate a list of predicted MST loci along with their read depths. This list of MST loci was used in the preparation of Fig. 1.

Code and data availability: All code used for this study will be made available upon request. Data from this study is available at the BioProject database using BioProject ID PRJNA283131.

## Results and Discussion

To verify the performance of our GME, we selected two colorectal cell lines: MST stable SW403 cells, and MST unstable DLD-1 cells^9^,^10^ (Extended Data Table 1). For each cell line, DNA isolated from cells grown as a single large cell culture was split and enriched using: (1) the Agilent exome enrichment kit; (2) our GME enrichment kit; or (3) an admixture of exome and our enrichment where the baits were combined prior to the enrichment. This enabled us to quantify the relative enrichment of repetitive regions for each enrichment system. Once enrichment performance was optimized and verified, we then sequenced 16 additional normal human DNA samples (Extended Data Table 1) from the 1000 Genomes Project using this enrichment system, and then processed the data to identify novel contigs that reproducibly appear in multiple samples. These concordant contigs were then analyzed for potential functional elements, as predicted by GlimmerHHM and supported by high similarity to RNA-Seq reads found in a variety of tissues. All enrichments, on average, had 99% of the high-quality reads map to the known human reference Hg19 (Table 1), consistent with what is expected given that 99% of the genome is “complete”. A substantial fraction of the reads from our GME enrichment, as opposed to those reads from exome enrichment, fell outside the exome (Table 1, Exome Overlap %), consistent with our goal of global enrichment for genomic microsatellite loci. On average, 0.45% of the total reads were found to be unmapped in the GME and the combined samples while only 0.1% of the exome enrichment samples were unmapped (Table 1). This is, again, consistent with the GME system specifically targeting repetitive regions. The GME of the DLD-1 cell line and the SW403 cell line were both enriched for microsatellite loci, but in different manners. DLD-1-GME captured a greater fraction of the known MSTs (identified from the Hg19 reference genome using Tandem Repeat Finder (TRF)^11^), whereas SW403-GME captured fewer of the known MSTs (Table 1) but had greater depth for a greater fraction of the MSTs that were captured (Extended Data Fig. 1). That this was due to variation in hybridization temperature is supported by an increase in C/G nucleotide sequences captured in this sample (Extended Data Fig. 2). While the MST loci amounts in the colorectal samples varied, the additional normal samples processed at the optimum temperature produced consistent results. On average, 87% of the mapped reads in the normal samples contained MST loci. (Table 1).

Any reads containing novel DNA/MST sequences are by definition unmapped relative to the reference genome, therefore we analyzed the content of the unmapped reads using our unmapped read analysis pipeline^8^ (and methods). We built contigs from those reads that did not align to the reference (Table 2), and then re-aligned these contigs to the reference to further eliminate any contigs with known sequence from further analysis. Not only were a higher number of contigs built from the unmapped read analysis on the GME samples, but there were also a higher number of contigs containing MSTs (Table 2), as expected. We identified between 162 and 1469 novel contigs per enrichment, of which between 8% and 56% contained MSTs (Table 2). These MST-containing loci were found to be covered at a significant read depth. On average, 65% of MSTs found in novel contigs of the normal samples and the colorectal cell lines were supported by more than 10 reads (Extended Data Figs 1 and 3). The analysis of all 20 independent GME samples (2 colorectal GME only, 2 colorectal GME combined with exome, and 16 normal) has yielded 790 concordant contigs (283 bp average length) that were observed in more than one sample (on average, each contig is seen in 5.6 samples, Extended Data Table 2). The concordant contigs were observed in as many as all 20 samples (Extended Data Table 3). This high reliability data (as confirmed in multiple samples) can reveal robust new MST-containing loci and high complexity sequence in unmapped regions. The distribution of the lengths of all the concordant contigs and those that contain a MST (Extended Data Fig. 4) shows that more than 90% of concordant contigs are shorter than 500 nt. As nextgen raw read lengths grow, longer contigs in these repeat-containing regions could be assembled.

Extended Data Fig. 5 shows that the GME approach can capture MST-containing loci of varying length; as short as 10 nt, but also longer than 150 nt (i.e. as observed in assembled contigs). Given that 14% of the known MSTs identified in Hg19 using TRF are >50 nt, our approach of enrichment plus contig building will, as raw read lengths grow, give access to longer MST loci.

To assess the potential value of these novel contigs, we searched the 790 concordant contigs for functional elements by aligning the contigs to 10 normal Lymphoblastoid RNA-Seq samples (Extended Data Table 4). We found that 37 concordant contigs aligned to at least one RNA-Seq read (Extended Data Table 5); and 22 of these 37 were supported by at least 10 RNA-Seq reads. BLASTing these 37 concordant contigs against the all known sequences in GenBank confirmed that the vast majority of these novel sequences (i.e. less than 50% identity to any portion of the known human reference) appear to be in human and chimpanzee subclones sequenced as part of the earliest human genome sequencing efforts, but not mapped to the current human genome reference, again confirming they are part of the missing human genome reference. This raises the possibility that sequence that had been relegated to the unaligned or pre-nextgen clone sequence trash heap may eventually be captured at a depth and with a reliability that allow them to be integrated into the reference. Table 3 illustrates the top 10 concordant contigs aligned to at least 235 RNA-Seq reads. Interestingly, the contig with the most hits to RNA-Seq reads was confirmed by BLASTing against GenBank to have 99% identity to abundant Ribosomal RNA sequences (45S, 60S, 28S) which may indicate a coding region for a new rRNA family member.

We also examined the 790 concordant contigs for the presence of gene-like structures (GLS) (e.g. putative exon, intron followed by another in-frame exon) using GlimmerHMM^12^ (Table 2). We found that 249 concordant contigs contained potential GLSs (Table 2). The majority of the contigs with GLS had 2–3 exons with no contig identified as having over 8 exons (Extended Data Table 6). Extended Data Fig. 4 shows that there was no significant difference in average contig length for contigs containing a MST and/or GLS. We then generated putative cDNAs from the concordant contigs identified as having GLS and compared them to 10 RNA-Seq data sets discussed above. Six of these putative cDNAs matched to the RNA-Seq data with between 1 and 90 RNA-Seq reads aligned to each GLS (Extended Data Table 7), demonstrating that these novel contigs not only have potential GLS, but some were identified as low abundance mRNAs. It further indicates that many new potential coding regions that robustly align to RNA-Seq reads contain functional elements not recognized by GlimmerHHM.

Analysis of the microsatellite motifs represented in the unmapped reads containing novel MSTs revealed an over-abundance of reads containing pentameric repeats (Fig. 1). A comparison of the relative abundance of the MST-containing contigs to the expected “known” motif ratios present from the human reference (Hg19) identified with Tandem Repeat Finder (TRF) and those present in a whole genome sequenced sample from the 1000 genomes project shows that the abundance of pentameric MST loci repeats are >3.5 fold more abundant in the whole genome sample than expected from the known reference (Fig. 1). Pentameric MSTs were present in the exome enrichment at a similar abundance as in the whole genome sequenced sample, however, in our GME the fraction of novel pentameric MSTs that were captured was an average of 9.2 fold greater than the fraction found in the known reference and 2.6 fold greater than the whole genome sequenced sample (a list of the top 5 most abundant motifs is given in Extended Data Table 8), consistent with these pentameric MSTs being localized extra-referentially.

Examination of the specific sequences of the MST containing loci revealed that the motifs AATGG and GTGGA were abundant in all samples, including the analysis of the whole genome sample, but not in the known human reference data (Extended Data Table 8). AATGG has been identified as a human centromeric^13^,^14^ sequence motif and therefore the overabundance of this sequence in the data, but its absence from the human reference assembly, is consistent with it being a component of centromeric heterchromatin. The motif GTGGA differs from AATGG by one nucleotide, and based on its similarity to a known centromeric repeat and overabundance in the data also makes it a candidate centromeric repeat as well. The overabundance of reads that contain the AATGG sequence was unexpected, and an average of 53% of unmapped reads with MSTs contained the AATGG motif, whereas, reads were devoid of the often-studied telomeric repeat, GGGTTA.

## Conclusion

Our GME provides the possibility for discovery of new/non-reference DNA sequence through the non-specific (i.e. not linked to a single reference locus) capture of repetitive DNA. Applying this target enrichment strategy broadly and pairing it with our algorithmic approach for identifying novel concordant contigs from unmapped reads can drive the human genome towards true completion; identify and annotate new potential functional elements therein; finish genomes containing even more repetitive sequence, such as plants; and be key to quantifying and comprehending the importance of telomeric and centromeric structure.

## Additional Information

**How to cite this article**: Fonville, N. C. *et al*. Genomic leftovers: identifying novel microsatellites, over-represented motifs and functional elements in the human genome. *Sci. Rep.* **6**, 27722; doi: 10.1038/srep27722 (2016).

## Supplementary Material

Supplementary Information

## Figures and Tables

**Table 1:** Mapping of enrichment reads.

Sample & Enrichment	Total Reads	Mapped %	Exome Overlap %	Mapped with MST %	Known MST Loci called %	Unmapped %	Unmapped with MST%
DLD-1- Exome	107763705	99.9	53.7	7.3	44.4	0.1	1.6
DLD-1 - GME	99869840	99.6	2.9	15.7	61.7	0.4	2.8
DLD-1 - Comb	86571454	99.8	52.3	12.1	41.1	0.2	4.2
SW403 - Exome	99392535	99.9	57.3	6.8	41.2	0.1	1.8
SW403 - GME	93042396	99.1	2.3	67.4	11.7	0.9	3.8
SW403 - Comb	95846052	99.7	51.7	12.0	27.3	0.3	3.3
Normal-1-GME	107184846	99.2	1.8	86.7	33.3	0.8	1.1
Normal-2-GME	86039208	98.4	1.9	87.5	26.3	1.6	0.5
Normal-3-GME	77776824	98.1	1.9	88.6	23.3	1.9	0.4
Normal-4-GME	76888422	99.0	2.0	89.1	23.1	1.0	0.9
Normal-5-GME	88088498	97.6	2.1	87.1	26.1	2.4	0.5
Normal-6-GME	87529722	97.7	2.0	87.7	25.9	2.3	0.4
Normal-7-GME	85362982	98.8	2.0	86.5	26.7	1.2	0.7
Normal-8-GME	69912104	98.3	1.8	88.7	24.1	1.7	0.6
Normal-9-GME	89072202	99.1	1.8	83.5	36.8	0.9	0.7
Normal-10-GME	88599848	99.1	1.9	87.0	30.6	0.9	0.7
Normal-11-GME	67477542	99.1	1.9	87.1	27.0	0.9	1.1
Normal-12-GME	74895624	98.4	2.1	87.6	27.3	1.6	0.5
Normal-13-GME	102597820	98.3	2.0	89.1	29.3	1.7	0.5
Normal-14-GME	39497576	98.5	1.8	89.6	22.4	1.5	0.6
Normal-15-GME	97582298	99.3	1.8	88.1	29.3	0.7	1.3
Normal-16-GME	91613940	98.1	1.8	86.7	32.0	1.9	0.6

**Table 2:** Novel contigs and MSTs from unmapped reads.

Sample & Enrichment	Novel Contigs	% Contigs with MSTs	% Contigs with GLS	% Contigs with both MST and GLS	Total MSTs	% Novel MSTs
DLD1-Exome	224	8	16	2	48	35
DLD1-GME	1469	20	23	7	510	71
DLD1-Comb	515	36	25	9	297	79
SW403-Exome	162	19	16	3	55	62
SW403-GME	372	46	34	19	227	92
SW403-Comb	267	38	34	15	153	88
Normal-1-GME	312	52	34	20	265	78
Normal-2-GME	278	55	28	18	244	82
Normal-3-GME	261	54	30	17	239	74
Normal-4-GME	289	53	30	17	255	75
Normal-5-GME	257	54	34	18	249	70
Normal-6-GME	316	50	33	17	253	79
Normal-7-GME	275	52	36	20	250	71
Normal-8-GME	255	56	31	18	219	82
Normal-9-GME	245	52	36	18	210	74
Normal-10-GME	221	54	31	17	197	75
Normal-11-GME	248	52	33	20	219	79
Normal-12-GME	265	51	38	21	221	76
Normal-13-GME	308	51	32	18	243	79
Normal-14-GME	198	52	29	16	192	68
Normal-15-GME	351	52	34	19	279	81
Normal-16-GME	307	51	28	16	254	79
Total	7395	42	29	14	5079	77
Concordant	790	42	32	16	533	100

**Table 3:** Alignment analysis of concordant contigs with normal lymphoblastoid RNA-Seq samples.

#	RNA-Seq samples										Total aligned reads	Contig length	BLAST hit
	1	2	3	4	5	6	7	8	9	10			
1	9441	17648	208	31	6957	15064	7134	2005	12173	7	70668	370	HS clone. Chr21
2	24	76	77	7	26	33	19	63	32	11	368	615	HS FOSMID clone. Chr7
3	24	67	75	7	21	33	20	61	32	11	351	628	HS FOSMID clone. Chr7
4	24	69	75	7	21	33	19	60	32	11	351	624	HS FOSMID clone. Chr7
5	55	38	28	11	4	48	0	48	46	26	304	531	HS FOSMID clone. Chr17
6	25	67	43	4	23	23	16	52	29	14	296	310	PT BAC clone. Chr7
7	18	25	38	5	57	36	35	7	30	24	275	303	HS BAC clone. Chr17
8	22	66	42	4	19	18	13	52	22	13	271	314	PT BAC clone. Chr7
9	21	66	41	4	16	18	13	51	21	13	264	323	PT BAC clone. Chr7
10	19	61	39	4	18	12	11	44	16	11	235	289	PT BAC clone. Chr7

The top 10 out of 37 concordant contigs that had RNA-Seq hits are presented in this table.

HS: Homo sapiens; PT: Pan troglodytes; Chr: chromosome.
