# Supplementary material for: Genomic leftovers: identifying novel microsatellites, over-represented motifs and functional elements in the human genome
Source: Sci Rep. 2016 Jun 9;6:27722. doi: 10.1038/srep27722 (PMC4899811; doi:10.1038/srep27722)

Genomic leftovers: identifying novel microsatellites, over-represented motifs and functional elements in the human genome

Natalie C. Fonville*, Karthik Raja Velmurugan*, Hongseok Tae, Zalman Vaksman, Lauren J. McIver, Harold R. Garner

Via Bioinformatics and Clinical Genetics Network, Edward Via College of Osteopathic Medicine, 2265 Kraft Dr, Blacksburg, VA 24060

*These authors contributed equally to this work.

Keywords: Microsatellite enrichment, next gen sequencing, unmapped reads, telomeric repeat, centromere, functional elements

Extended Data Table 1
Details of the samples on which GME was performed.

Sample #	Sample	Sample Type	Sample Source
1	DLD1-Exome	Colorectal	ATCC CCL-221
2	DLD1-GME	Colorectal	ATCC CCL-221
3	DLD1-Comb	Colorectal	ATCC CCL-221
4	SW403-Exome	Colorectal	ATCC CCL-230
5	SW403-GME	Colorectal	ATCC CCL-230
6	SW403-Comb	Colorectal	ATCC CCL-230
7	Normal-1-GME	Lymphoblastoid	Coriell HG00384
8	Normal-2-GME	Lymphoblastoid	Coriell HG00383
9	Normal-3-GME	Lymphoblastoid	Coriell HG00382
10	Normal-4-GME	Lymphoblastoid	Coriell HG00381
11	Normal-5-GME	Lymphoblastoid	Coriell HG00380
12	Normal-6-GME	Lymphoblastoid	Coriell HG00379
13	Normal-7-GME	Lymphoblastoid	Coriell HG00378
14	Normal-8-GME	Lymphoblastoid	Coriell HG00377
15	Normal-9-GME	Lymphoblastoid	Coriell HG00376
16	Normal-10-GME	Lymphoblastoid	Coriell HG00375
17	Normal-11-GME	Lymphoblastoid	Coriell HG00373
18	Normal-12-GME	Lymphoblastoid	Coriell HG00372
19	Normal-13-GME	Lymphoblastoid	Coriell HG00371
20	Normal-14-GME	Lymphoblastoid	Coriell HG00369
21	Normal-15-GME	Lymphoblastoid	Coriell HG00368
22	Normal-16-GME	Lymphoblastoid	Coriell HG00367

ATCC: American Type Culture Collection

Extended Data Table 2

The statistics of the concordant contigs are furnished in the below table. The data is divided according to the number of mismatches allowed for the concordant contig calculation to illustrate that if some mismatch is allowed (due to possible variations among individuals) the additional contigs may be further assembled. However, for further analysis, only those concordant contigs assembled using the strict 0 mismatch value were used.

Statistics	Mismatch	
	0	1
Total concordant contigs (observed in at least 2 samples)	790	747
Total sample contigs found concordant	4419	4915
Maximum # of sample contigs assembled into a concordant contig	25	29
Minimum # of sample contigs assembled into a concordant contig	2	2
Average # of samples a concordant contig was found	5.6	6.6

Extended Data Table 3. Concordant contig sample distribution

Novel contigs that were present in multiple samples are considered to be concordant. The table groups the concordant contigs according to the number of samples in which they were found. The data is divided by mismatch 0 and mismatch 1 (Number of mismatches allowed while generating the concordant contigs).

# of samples	Concordant contigs	
	0mismatch	1mismatch
2	243	188
3	125	99
4	85	80
5	59	48
6	36	50
7	44	38
8	29	30
9	31	28
10	20	24
11	26	30
12	14	21
13	18	29
14	13	17
15	9	13
16	8	11
17	8	9
18	15	18
19	7	11
20	0	2

Extended Data Table 4

Details about the RNA-Seq samples that were used to confirm that the concordant contigs contain potential functional elements.

Sample #	SampleID	Sample Type	Sample Source
1	ERR188040	Lymphoblastoid	ArrayExpress - EBI
2	ERR188231	Lymphoblastoid	ArrayExpress - EBI
3	ERR188043	Lymphoblastoid	ArrayExpress - EBI
4	ERR188280	Lymphoblastoid	ArrayExpress - EBI
5	ERR188325	Lymphoblastoid	ArrayExpress - EBI
6	ERR188327	Lymphoblastoid	ArrayExpress - EBI
7	ERR188373	Lymphoblastoid	ArrayExpress - EBI
8	ERR188313	Lymphoblastoid	ArrayExpress - EBI
9	ERR188382	Lymphoblastoid	ArrayExpress - EBI
10	ERR188359	Lymphoblastoid	ArrayExpress - EBI

Extended Data Table 5:

Thirty-seven concordant contigs aligned to at least one RNA-Seq read. As indicated in methods, a RNA-Seq hit must align with 0 mismatches to at least 70% of the length of the contig.

#	RNA-Seq samples										Total aligned reads	Contig length	BLAST hit
	1	2	3	4	5	6	7	8	9	10			
1	9441	17648	208	31	6957	15064	7134	2005	12173	7	70668	370	HS clone. Chr21
2	24	76	77	7	26	33	19	63	32	11	368	615	HS FOSMID clone. Ch
3	24	67	75	7	21	33	20	61	32	11	351	628	HS FOSMID clone. Ch
4	24	69	75	7	21	33	19	60	32	11	351	624	HS FOSMID clone. Ch
5	55	38	28	11	4	48	0	48	46	26	304	531	HS FOSMID clone. Ch
6	25	67	43	4	23	23	16	52	29	14	296	310	PT BAC clone. Chr7
7	18	25	38	5	57	36	35	7	30	24	275	303	HS BAC clone. Chr17
8	22	66	42	4	19	18	13	52	22	13	271	314	PT BAC clone. Chr7
9	21	66	41	4	16	18	13	51	21	13	264	323	PT BAC clone. Chr7
10	19	61	39	4	18	12	11	44	16	11	235	289	PT BAC clone. Chr7
11	21	61	37	4	18	11	9	43	15	11	230	294	PT uncharacterized
12	14	54	38	2	13	13	8	37	14	11	204	274	PT uncharacterized
13	5	11	37	2	67	4	3	7	0	2	138	327	HS clone.
14	11	18	2	0	66	13	3	12	10	0	135	242	HS rRNA gene
15	13	15	8	1	13	11	0	27	18	9	115	387	HS clone. Chr17
16	11	18	13	0	9	7	6	12	11	8	95	466	HS clone. Chr21
17	5	8	8	0	28	0	2	9	3	9	72	363	HS FOSMID.
18	7	13	13	0	22	6	1	3	1	5	71	285	PT BAC clone. ChrY
19	1	3	9	2	14	0	0	2	0	1	32	437	PA BAC clone. Chr16
20	3	11	4	0	2	0	0	6	2	1	29	324	HS clone. Chr21
21	0	2	0	6	0	0	2	8	3	0	21	1016	BB genome scaffold
22	1	5	1	4	0	0	0	1	0	3	15	512	No hits
23	0	0	1	0	1	1	0	3	0	3	9	370	HS clone. Chr21
24	1	2	0	2	0	0	0	0	2	0	7	368	HS FOSMID clone. Ch
25	0	0	1	0	1	0	0	2	0	2	6	372	HS clone. Chr21
26	1	0	2	0	0	0	0	2	0	0	5	423	HS BAC clone.
27	1	1	0	0	3	0	0	0	0	0	5	495	HS clone. Chr9
28	0	0	0	0	3	2	0	0	0	0	5	346	HS clone. Chr21
29	0	0	0	0	0	0	0	3	0	0	3	381	PT BAC clone. Chr7
30	1	0	0	0	1	0	0	0	0	0	2	259	OF genome scaffold
31	0	0	0	0	0	0	0	0	0	1	1	449	PT BAC clone. ChrY
32	0	0	0	0	0	0	0	1	0	0	1	314	HS FOSMID clone. Ch
33	0	0	1	0	0	0	0	0	0	0	1	232	No hits
34	0	0	1	0	0	0	0	0	0	0	1	364	No hits
35	0	1	0	0	0	0	0	0	0	0	1	277	HS contig.
36	0	1	0	0	0	0	0	0	0	0	1	283	HS clone. ChrX
37	0	0	0	0	0	0	0	0	1	0	1	295	HS clone. Chr17

HS: Homo sapiens; PT: Pan troglodytes; BB: Babesia bigemina; OF: Onchocerca flexuosa; Chr: Chromosome.

Extended Data Table 6

Distribution of predicted exon number within the concordant contigs predicted by GlimmerHMM to have Gene-Like Structures (GLS).

#Exons	#GLS
2	73
3	105
4	44
5	24
6	2
8	1
Total	249

Extended Data Table 7:
Six putative cDNAs found in the concordant contigs had at least one read hit in the RNA-Seq samples.

#	Aligned RNA-Seq reads	Contig length	cDNA length	BLAST hit
1	90	314	219	PT BAC clone. Chr7
2	10	323	108	PT BAC clone. Chr7
3	5	495	384	HS clone. Chr9
4	1	370	159	HS clone. Chr21
5	1	259	138	OF genome scaffold
6	1	368	159	HS FOSMID clone. Chr11

PT: Pan troglodytes; HS: Homo sapiens; OF: Onchocerca flexuosa; Chr: Chromosome.

Extended Data Table 8. Top five pentameric and hexameric motif families. The top five pentameric and hexameric motif families are shown for the DLD-1 and SW404 samples, a representative split (50,000 unmapped reads) from a whole genome sequenced sample and the known human reference hg19 where MSTs were identified using Tandem Repeat Finder (Hg19-TRF). The telomeric repeat is underlined.

	DLD-1 Exome			DLD-1 GME			DLD-1 Combined		
	Motif	Loci	Percent	Motif	Loci	Percent	Motif	Loci	Percent
Pentamer	AATGG	2	22	AATGG	20	20	GTGGA	118	47.2
	ATATA	2	22	GTGGA	20	20	AATGG	116	46.4
	AGGGG	2	22	ATATA	6	6	GAATT	1	0.4
	GGGAT	1	11	GGGGC	4	4	GGATT	1	0.4
	TTTTC	1	11	GCCCT	4	4	GGGAT	1	0.4
Hexamer	TCCTCT	1	25	CATCAC	4	40	CCCCGG	1	25
	GGGGGA	1	25	CCCTCA	2	20	AGGTCC	1	25
	CAGCAA	1	25	GGCCCA	2	20	CCTGGC	1	25
	CCTGGC	1	25	ATAAAA	2	20	CCCTCA	1	25

	SW404 Exome			SW403 GME			SW403 Combined		
	Motif	Loci	Percent	Motif	Loci	Percent	Motif	Loci	Percent
Pentamer	GTGGA	8	36.4	AATGG	107	55.7	AATGG	57	48.3
	AATGG	5	22.7	GTGGA	64	33.3	GTGGA	55	46.6
	ATATA	2	9.1	CCCAC	11	5.7	CCCAC	2	1.7
	CAAAA	1	4.5	ATTCG	2	1	GGATT	1	0.8
	AGGGG	1	4.5	CCTTC	1	0.5	ATTTT	1	0.8
Hexamer	TCCTCT	1	33.3	CAGCAA	1	20	CTGGGG	1	16.7
	GGCCCA	1	33.3	CAACGA	1	20	TCCTCT	1	16.7
	CCTGGC	1	33.3	CTGGGG	1	20	CCCCGG	1	16.7
				AAAATG	1	20	AGGGTC	1	16.7
				CCCTCA	1	20	AAAATG	1	16.7

	HG19 TRF			Whole Genome		
	Motif	Loci	Percent	Motif	Loci	Percent
Pentamer	CAAAA	51763	28	AATGG	2192279	82.1
	ATTTT	39451	21.3	CAAAA	159237	6
	TTTTC	26684	14.4	ATTTT	89791	3.4
	ATTAA	6162	3.3	TTTTC	52059	2
	CTTTC	4678	2.5	GTGGA	50117	1.9
Hexamer	ACAAAA	20644	17	ACAAAA	41995	25.9
	TCTTTT	16995	14	ATAAAA	21847	13.5
	ATAAAA	16293	13.4	GGGTTA	14626	9
	GTATAT	4297	3.5	TCTTTT	13237	8.2
	CATACA	3557	2.9	TGTCTC	4982	3.1

Extended Data Figure 1. Read coverage analysis for MST motifs. For every sample >15% of known microsatellite loci (MSTs in mapped reads) (A) and >50% of novel microsatellite loci (MSTs in novel contigs) (B) were covered by more than 10 sequencing reads.

A

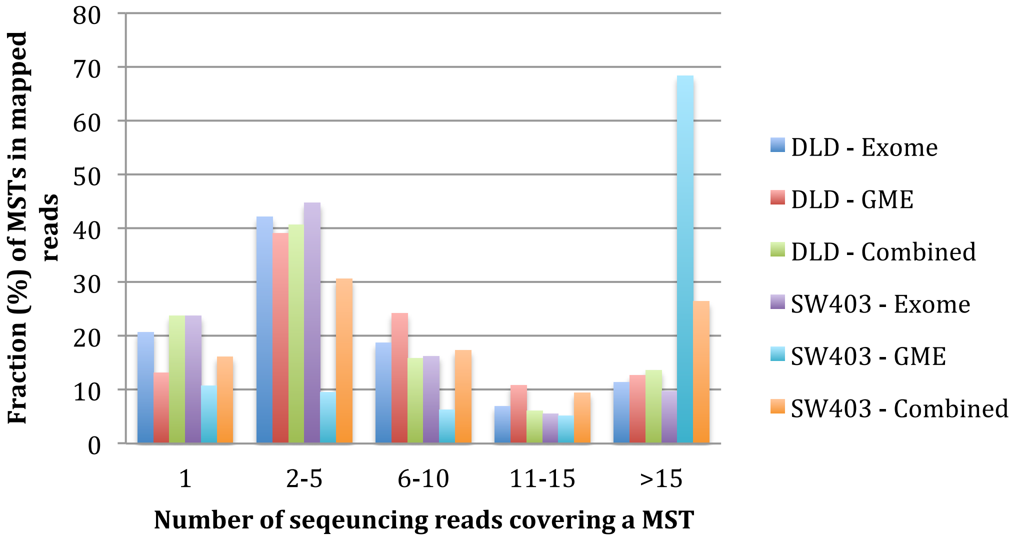

B

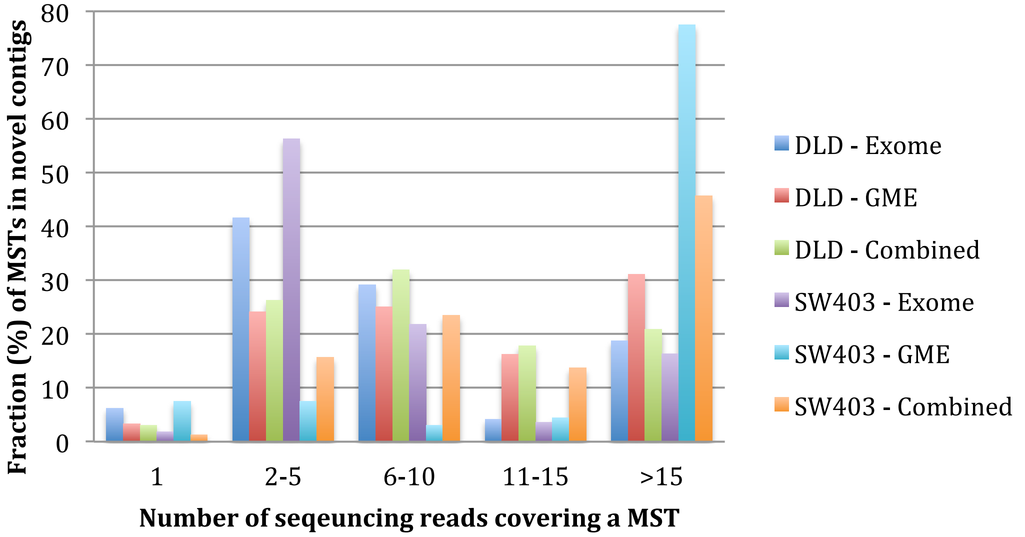

Extended Data Figure 2. Homopolymer Nucleotide Distribution

As our GME captures repetitive motifs based on 30nt repetitive regions, it was possible that C/G nucleotides were captured in higher abundance than with standard exome capture. We found that the SW403-GME sample, but not the DLD1-GME enriched sample was enriched for C/G nucleotides. This difference in capture between the two GME samples may be due to slight differences in hybridization temperatures used while optimizing the capture process, and may indicate a use for this technology in specifically capturing C/G nucleotide regions in the future.

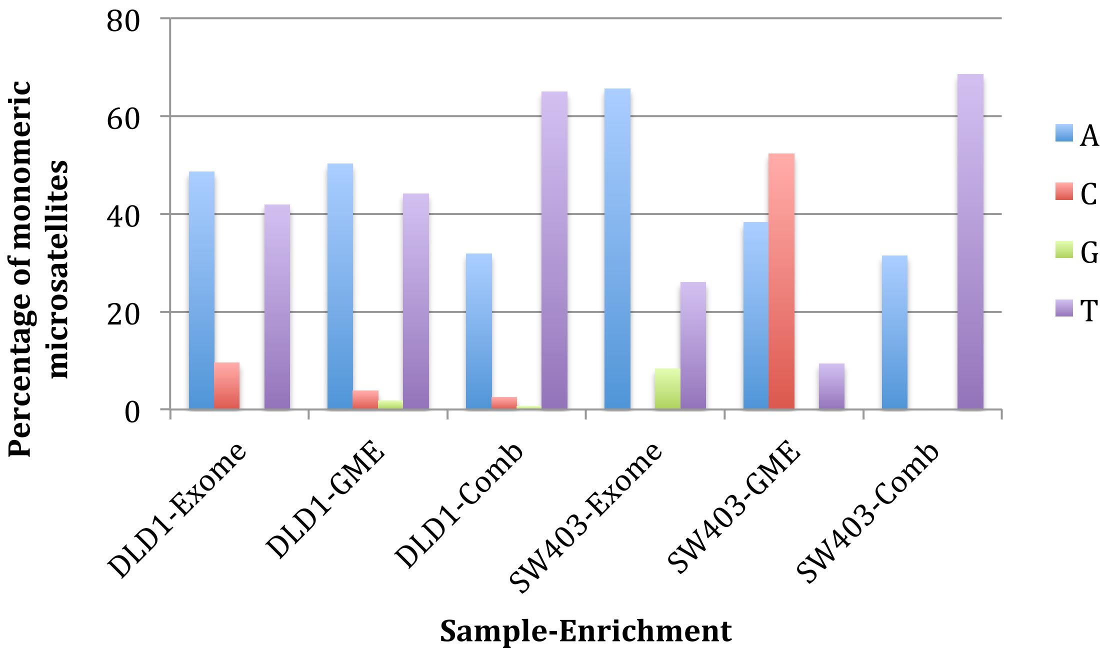

Extended Data Figure 3. Read coverage analysis of MST motifs in normal human samples. (A) In every individual sample more than 30% of the MSTs are covered by more than 15 reads while an equal percentage are covered by 2 to 5 reads. (B) Approximately 60% of MSTs found in the novel contigs are covered by more than 15 reads.

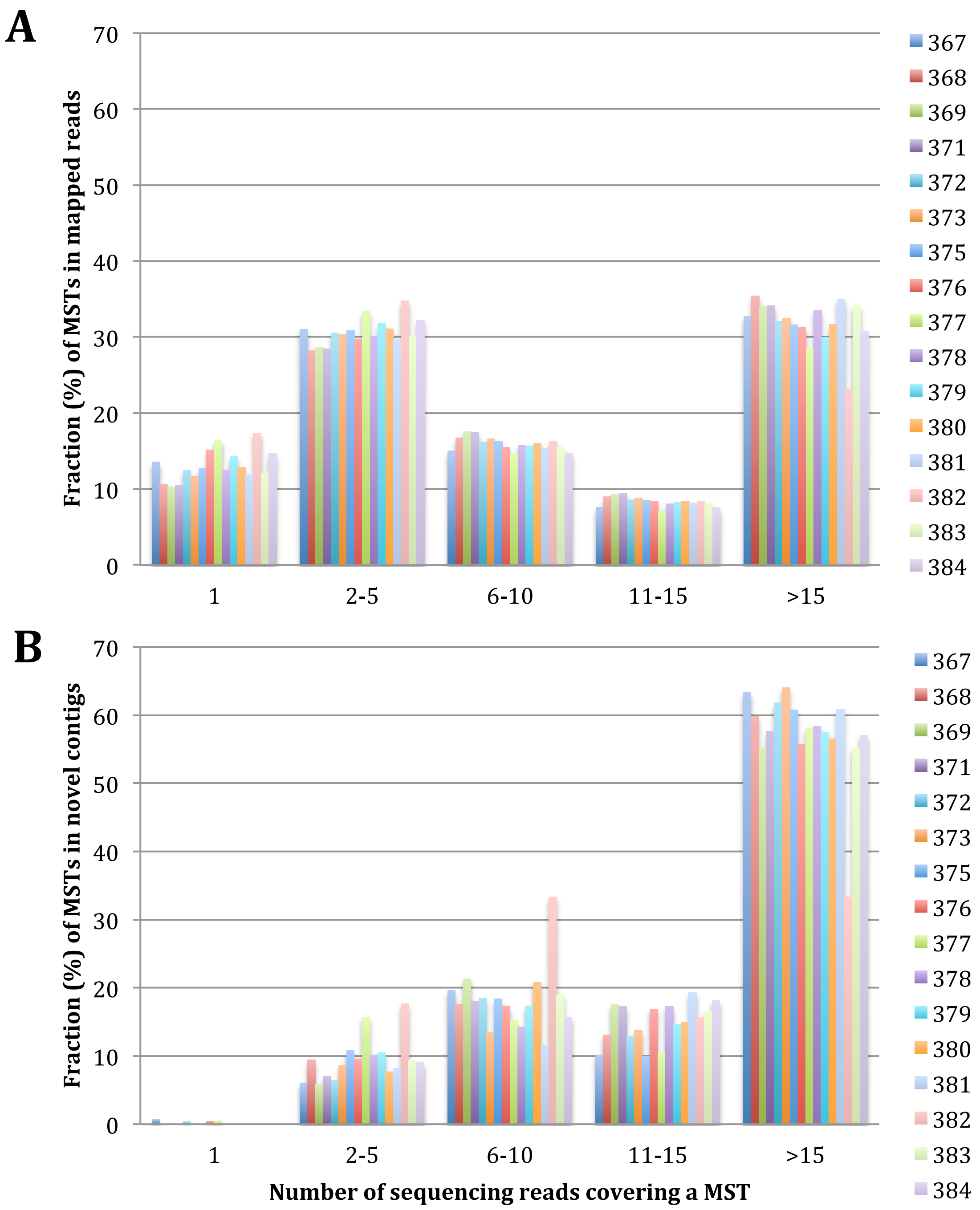

Extended Data Figure 4. Concordant contig length distribution
This histogram shows how the concordant contigs with and without MSTs and GLS are distributed according to their lengths. Each category of contigs represents the combined information of all the samples (i.e. 16 GME sequenced normal samples, 3 DLD1 samples and 3 SW403 samples). It should be noted that except for the "less than 250 bases" bin, all other contigs contain two or more reads.

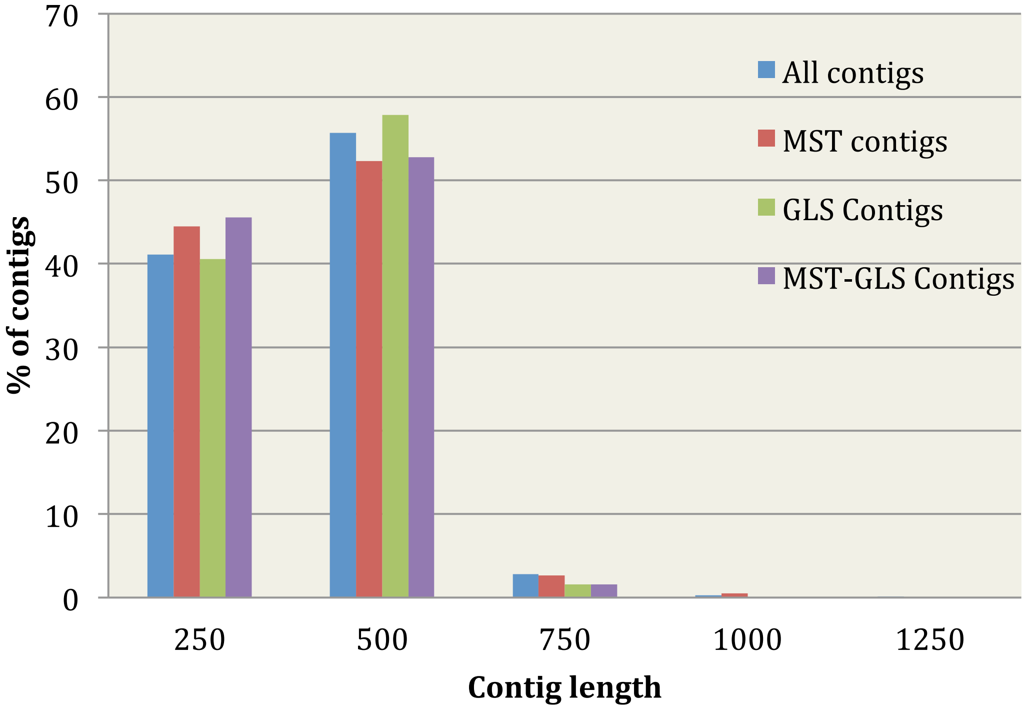

Extended Data Figure 5. Length distribution of novel microsatellites.
The following histograms show the MSTs according to their length in the colorectal cell lines (A) and in the normal samples (B).

A

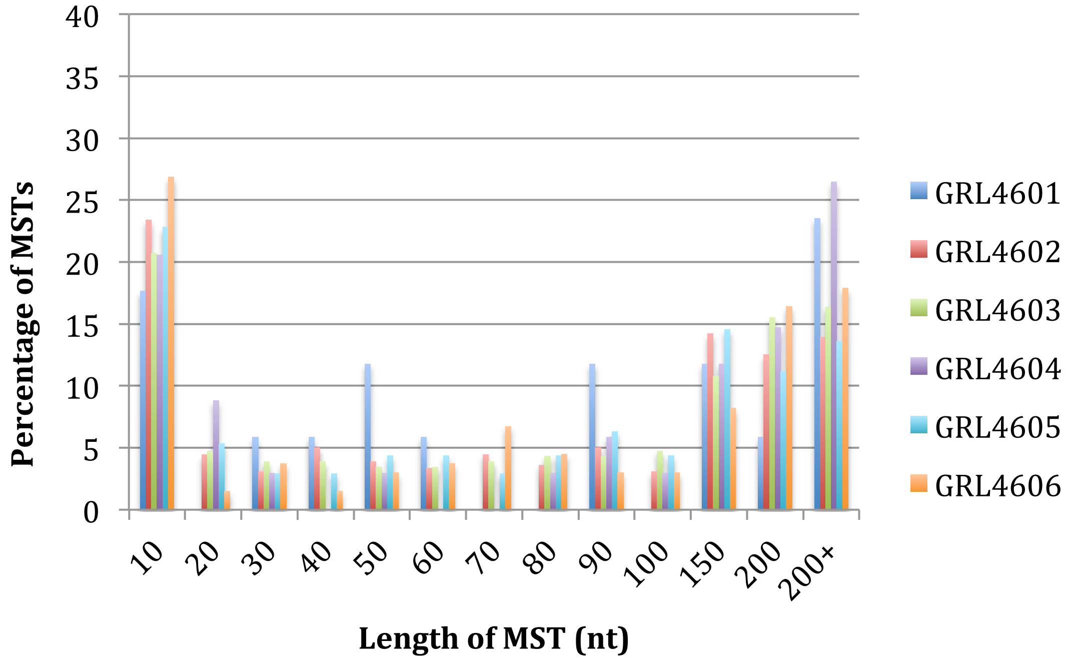

B

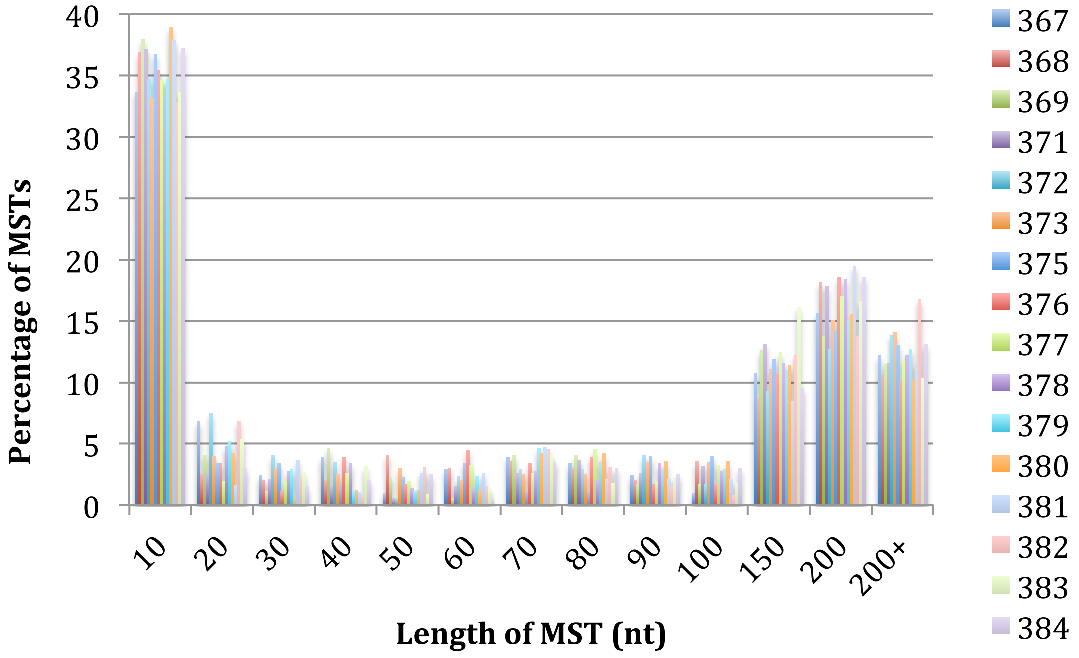

Supplement: Supplementary Information [file srep27722-s1.pdf]
